# Practical Remarks upon Some Important Medicinal Agents Not in General Use

**Published:** 1854-02

**Authors:** F. E. Wilkinson


*Practical Remarks upon some Important Medicinal Agents not in General Use.* By F. E. Wilkinson, M. D.

*Strychnia.*—Dr. Marshall Hall, in some recent papers published in *The Lancet,* speaks very highly of the beneficial effects of minute doses of strychnia, dissolved in acetic acid. I have for some years been in the habit of prescribing small doses of strychnia, and can corroborate Dr. Hall's valuable testimony in every particular; therefore any remarks of mine, upon its effects in epileptic cases, would be unnecessary.

My method has been to dissolve two grains of strychnia in one ounce of phosphoric acid of the London Pharmacopœia—a very speedy and certain solvent, and also possessing, doubtless, the additional excellent property of assisting the good effect of the strychnia upon the brain and nervous system. Of this solution, in cases of prolonged dyspepsia, in neuralgia, indeed in many states of the nervous system requiring tone, it has been usual with me to administer, after the state of the secretions have been attended to, a dose, consisting of five minims of the above solution, three or four times daily, either alone or combined with some other appropriate remedy, according to the nature and complication of the case. This medicine has also a remarkable effect in ague. During a four years' residence at Cambridge, I had opportunities of seeing the treatment of a vast number of cases of intermittent fever, but in no one instance, as far as my memory serves me, was recovery nearly so rapid as I have seen it after the administration of this powerful remedy.

I subjoin a few cases, illustrating these remarks, whilst I know many friends who could readily corroborate these facts from cases occurring in their practices.

*Neuralgia.*—July 11, 1851.—Mrs. P——, married, aged thirty-two, pale and emaciated. She has been for some years the subject of neuralgia, which attacks her whenever she takes cold, and occasionally quite incapacitates her from attending to the duties of her family. She is now suffering acutely from pain and the loss of rest. The bowels being confined, the following was ordered:—Calomel three grains; conserve of roses, sufficient quantity to make into a pill. Liquor of strychnia, (two grains to an ounce,) twenty minims; sulphate of magnesia, one ounce; spearmint water, six ounces. To take one-fourth three times a day.

12th.—The pain seems relieved this afternoon, but the patient is still suffering from loss of rest. To take belladonna, a quarter of a grain; extract of hyoscyamus, four grains; mix, and make into a pill.

13th.—Slept well; feels better. The medicine has acted too freely upon the bowels.

14th.—Is quite free from pain, but still a numbness exists in the old place; continue the mixture.

15th.—The pain has entirely left; feels more cheerful, and free from pain than she has done for many months; appetite improving.

From this time the patient continued free from pain, and gradually recovered a great amount of health and strength under the continued use of the above medicine, combined latterly with some tincture of iron.

Case 2.—1851,—C. P——. In this case the disease had been existing more or less constantly, the pain scarcely leaving her entirely for some months. Is now suffering from bilious diarrhœa. After prescribing a dose of calomel and opium, followed by a rhubarb purge, to cleanse away irritating matter, I prescribed iodide of potassium, two grains; liquor of strychnia, five minims; water one ounce, three times a day. After taking the medicine for some days the pain entirely ceased. The treatment was continued for six weeks, and she has had no return of the symptoms for now nearly three months.

*In Ague Cases.*—Jan. 5th, 1852.—J. W.——was taken ill yesterday, just before leaving work, with violent pain in the left side, and with uncontrollable shivering, followed by fever, with some amount of delirium, and then by profuse perspiration. Has not felt quite well for some time. Is suffering from the cold stage of intermittent at the present time; looks blue and pinched. Ordered, calomel, three grains; Dover's powder ten grains; to be made into a powder and to be taken immediately. A compound jalap powder in the morning. Slept pretty well: medicine acted freely. Shivering returned in the morning. Altogether the patient does not think himself improved. Liquor of strychnia, forty minims; water, six ounces; to be made into a mixture; one-forth to be taken every four hours.

7th.—The time of accession of the paroxysm was later and the attack less violent.

8th.—Continues improving.

From this time the patient gradually improved, the dose of strychnia being gradually diminished, and on calling to see him on the 14th, he had returned to work, feeling himself quite well.—*Lancet.*

